# Apoptosis Transcriptional Profile Induced by *Porphyromonas gingivalis* HmuY

**DOI:** 10.1155/2019/6758159

**Published:** 2019-03-18

**Authors:** Paulo C. Carvalho-Filho, Lilia F. Moura-Costa, Ana C. M. Pimentel, Mabel P. P. Lopes, Sibelle A. Freitas, Patrícia M. Miranda, Ryan S. Costa, Camila A. V. Figueirêdo, Roberto Meyer, Isaac S. Gomes-Filho, Teresa Olczak, Márcia T. Xavier, Soraya C. Trindade

**Affiliations:** ^1^Department of Immunology, Federal University of Bahia, Bahia, Brazil; ^2^Dental School, Bahiana School of Medicine and Public Health, Brazil; ^3^Department of Periodontics, Feira de Santana State University, Bahia, Brazil; ^4^Laboratory of Medical Biology, Faculty of Biotechnology, University of Wroclaw, Wroclaw, Poland

## Abstract

This study aimed at evaluating the transcriptional profile of apoptosis-related genes after *in vitro* stimulation of peripheral blood mononuclear cells (PBMCs) derived from individuals with periodontitis (P) and healthy nonperiodontitis (NP) control subjects with *P. gingivalis* HmuY protein. PBMCs from the P and NP groups were stimulated with HmuY *P. gingivalis* protein, and the expression of genes related to apoptosis was assessed by custom real-time polymerase chain reaction array (Custom RT^2^ PCR Array). Compared with the NP group, the P group showed low relative levels of apoptosis-related gene expression, downregulated for FAS, FAS ligand, TNFSF10 (TRAIL), BAK1, CASP9, and APAF1 after *P. gingivalis* HmuY protein stimulation. Furthermore, the P group exhibited low levels of relative gene expression, downregulated for CASP7 when the cells were not stimulated. Our data suggest that *P. gingivalis* HmuY protein might participate differently in the modulation of the intrinsic and extrinsic apoptosis pathways.

## 1. Introduction

Periodontitis is a multifactorial disease, with significant participation of the host, environmental factors, and bacterial components. It is known that keystone pathogens, such as *Aggregatibacter actinomycetemcomitans*, *Porphyromonas gingivalis*, *Tannerella forsythia*, and *Treponema denticola*, in the subgingival biofilm elicit a host inflammatory response, which can lead to periodontal breakdown. [1]. The microbial diversity of the oral cavity is immense, and the host response during periodontitis is complex, with components of the innate and adaptive immune system that lead to chronic inflammation and bone resorption [2].

Recent metagenomic and mechanistic studies are consistent with a new periodontal pathogenesis model that proposes that periodontal diseases are caused by a synergistic and dysbiotic microbial community, not by a selected group of bacteria known as “periodontopathogens”. Bacteria found in low abundance in the microbiota have an effect throughout the community and are critical components for the development of dysbiosis. They are known as “keystone” pathogens [3]. However, an increased abundance in these known pathogens is observed, related to the presence as well as the severity of the disease, indicating a microbial variation in the dysbiotic process [4].

Virulence factors of *P. gingivalis*, the main keystone pathogen in periodontitis, can determine a great immunogenicity to stimulate innate and adaptive immune host responses. Among them are capsule components, lipopolysaccharide (LPS), fimbriae, and outer membrane proteins, such as associated and secreted gingipains. Gingipains specific for arginine (HRgpA and RgpB) and lysine (Kgp) play an important role in the infection, supporting the onset and progression of periodontitis [5].

*P. gingivalis* can invade the periodontal tissues and use a panel of virulence factors to evade the innate immune and inflammatory responses [6]. It has been shown that this pathogen has the ability to invade epithelial cells [7], which can be an escape mechanism from host defenses, favoring the penetration of the microorganism into the bloodstream and thus acting systemically in the human body [8].

*P. gingivalis* ability to obtain nutrients and evade the host immune response in the microenvironment is directly related to its survival, proliferation, and infection. One of the essential nutrients for its development is iron. This component is most abundant in the host in the form of heme [9]. The heme capture can be performed by a *P. gingivalis* HmuY, which sequesters and delivers heme to the outer membrane receptor HmuR [10–12].

There is a syntrophy among the species of the oral biofilm, revealing the interspecies cooperation in the acquisition of nutrients [13]. This mechanism reveals how HmuY works together with proteases produced also by other bacterial species to acquire heme from hemoglobin and may represent mutualism between *P. gingivalis* and *Prevotella intermedia* cohabiting the periodontal pocket [14]. However, HmuY is recognized by highly specific antibodies, suggesting that this protein can serve as a specific antigen of this bacterium [15].

*P. gingivalis* HmuY also seems to act in the programmed cell death process. Human peripheral blood mononuclear cells (PBMCs) cultured with the protein appear to be unable to complete the process of apoptosis, resulting in death characterized by the release of inflammatory cell content in the microenvironment, such as late apoptosis and necrosis, which can prolong the tissue destruction process [16]. This protein induces high levels of Bcl-2 in the mononuclear cells of individuals with periodontitis, resulting in the inhibition of apoptosis and thereafter enabling increased survival of CD3^+^ T cells, which prolong the chronic inflammatory condition in periodontitis [17].

The modulation of cell death molecules and mechanisms evolved by periodontopathogens during the establishment of periodontitis has been demonstrated [18–21]. However, the apoptosis of mononuclear cells under bacterial challenge is unclear. The present study identifies specific *P. gingivalis* components that are involved in these events, which is the novelty of the study. In addition, the role of HmuY in triggering the extrinsic and intrinsic apoptosis pathways is not well understood. Thus, this study was carried out to evaluate the transcriptional profile of genes involved in apoptosis mechanisms in PBMC under *P. gingivalis* HmuY protein stimulation.

## 2. Materials and Methods

### 2.1. Participants

The present study was approved by the Feira de Santana State University Institutional Review Board (No. 0203.0.059.000-11). All volunteers in this study signed the free and informed consent form. A total of 8 individuals with severe periodontitis (P group) and 8 nonperiodontitis controls (NP group) were recruited between 2013 and 2014 from the College of Dentistry at the Feira de Santana State University, Bahia, Brazil. Volunteers with diabetes, pregnancy, smoking habit, any autoimmune disease, cardiovascular disease, prior periodontal treatment, and anti-inflammatories and antibiotics used within two and six months before inclusion in this study, respectively, were the exclusion criteria.

### 2.2. Disease Classification

A dentist (P.C.C.F.) was trained in the calibration process for periodontal examination (kappa interexaminer agreement value = 0.932) using a Williams periodontal probe (Hu-Friedy, Chicago, IL, USA). The following clinical parameters were evaluated: probing depth (PD), clinical attachment level (CAL), and bleeding on probing (BOP).

Participants were diagnosed as having periodontitis (P group) if they had at least four teeth with at least one site with a probing depth greater than or equal to 4 mm, clinical attachment loss of 3 mm or more, and bleeding on probing at the same site [22]. All individuals with periodontitis had a diagnosis of severe form of the disease, since they had at least 2 interproximal sites with clinical attachment loss greater than or equal to 6 mm (not affecting the same tooth) and at least 1 interproximal site with probing depth greater than or equal to 5 mm [23].

Those participants who did not meet these criteria were considered not to have periodontitis (NP group).

### 2.3. Antigen

The *P. gingivalis* HmuY protein (NCBI ID number: CAM 31898) was overexpressed, purified, and lyophilized as described previously [24] and subsequently dissolved in PBS to a final concentration of 2.5 *μ*g/mL.

### 2.4. Blood Collection and Cell Culture

Peripheral venous blood (20 mL) was collected from the volunteers in heparinized tubes. Peripheral blood mononuclear cells (PBMCs) were obtained by density centrifugation in accordance with the manufacturer's guidelines (SepCell, StemCell Technologies Inc., USA) and then cultured in flat-bottom 24-well plates (10^6^ cells/well) in RPMI (Roswell Park Memorial Institute) medium (LGCBio, São Paulo, SP, Brazil), containing 10% fetal calf serum (inactivated by heat) and 1% antibiotic/antimycotic drug (R&D Systems, Minneapolis, MN, USA). Cells (10^6^ per well) were incubated with 5 *μ*g/mL of pokeweed mitogen (PWM), 2.5 *μ*g/mL of *P. gingivalis* HmuY protein, or in the absence of antigens (nonstimulated cells), under 5% CO_2_ in humid conditions for 48 h at 37°C.

### 2.5. Gene Expression Analysis

RNA was extracted from the cells using a miRNeasy Mini Kit (SABiosciences Corp.) in accordance with the manufacturer's instructions. cDNA was synthesized using 500 ng of the total RNA and a RT^2^ First Strand Kit (SABiosciences Corp.).

The Custom Human RT^2^ Profiler PCR Array: CAPH12794 (SABiosciences Corp.), designed for this study, was used for the sample analysis. Altogether, 45 different genes were simultaneously amplified in the sample using 384-well plates. To verify the presence of a single amplicon, a melting curve was performed.

PCR arrays were carried out using Applied Biosystems QuantStudio 12K Flex Real-Time PCR System 384-well block (Applied Biosystems). 10 *μ*L of a mixture composed of 2× SABiosciences RT^2^ qPCR Master Mix and cDNA was applied into each well. The Ct values were calculated for each gene using the Applied Biosystems software. Baseline and threshold measurements were determined according to the manufacturer's guidelines (SABiosciences, Qiagen).

### 2.6. Statistical Analysis

The software supplied by Qiagen (https://www.qiagen.com/br/shop/genes-and-pathways/data-analysis-center-overview-page) identified glucuronidase beta (GUSB) as the most stably expressed housekeeping gene for data normalization. The fold change in gene expression (compared to positive control – PBMC – P group) was calculated using the ΔΔCt method. A more than twofold change in gene expression (compared to positive control – PBMC – P group) was considered as the up- or downregulation of a specific gene expression.

The RT^2^ Profiler PCR Array Data Analysis software does not perform any statistical analysis beyond the calculation of *p* values using Student's *t*-test based on 2^(-ΔCT) values for each gene P group compared to the NP group. The Microarray Quality Control (MAQC) published results indicating that a ranked list of genes based on fold change and such a *p* value calculation was sufficient to demonstrate reproducible results across multiple microarray and PCR Arrays including the RT^2^ Profiler PCR Arrays [25].

## 3. Results

The comparison between the periodontitis (P) and nonperiodontitis (NP) groups demonstrated that there were no statistically significant differences in the sex (*p* = 0.96) or age (*p* = 0.57) (Table 1). On the other hand, periodontal status was worse in the periodontitis individuals, who showed a higher percentage of teeth with bleeding on probing (*p* = 0.03), probing depth ≥ 4 mm (*p* = 0.001), and clinical attachment loss ≥ 3 mm (*p* = 0.001).

### 3.1. Expression Profile of Apoptosis Gene Targets: Heat Map

**Figure 1:**
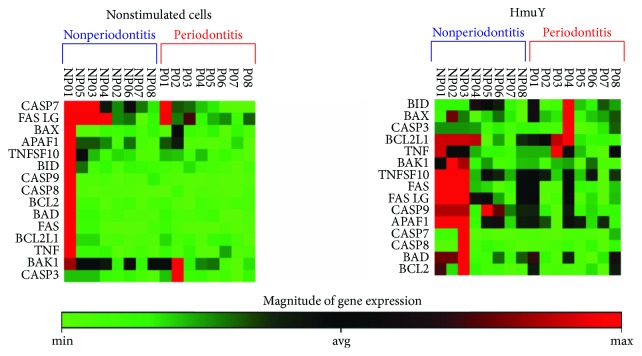
Gene cluster identification involved in apoptosis mechanisms by PBMC from individuals with periodontitis (P) and nonperiodontitis (NP) under *P. gingivalis* HmuY protein (HmuY) stimulus and in the absence of stimulus (nonstimulated cells). The green-black-red color gradient represents relative levels of gene expression, indicating under-even-over regulation, respectively.

The analysis of the mRNA expression pattern indicated differences between the P and NP groups both in cells without stimulation (nonstimulated cells) and under *P. gingivalis* HmuY protein stimulus (HmuY). The variability in the pattern of global gene expression between the P and NP samples indicated heterogeneity, particularly in those stimulated with HmuY protein. Under both conditions of culturing, stimulation with HmuY (HmuY) and without antigenic stimulation (nonstimulated cells), PBMC of individuals of the NP group showed a higher number of genes upregulated, and on the other hand, individuals with periodontitis showed a downregulated gene expression profile. Furthermore, differences were more evident in the NP and P groups when stimulated with *P. gingivalis* HmuY protein (HmuY), as can be observed in Figure 1.

In nonstimulated cells, the expression of CASP7 and FASLG was upregulated in a higher number of individuals of the NP group (3 and 4 individuals, respectively). Patient NP1, without the diagnosis of periodontitis, showed upregulation in most of the genes examined. In the P group, the upregulation of CASP7, FAS, BAK, and CASP3 occurred in one case for each gene and in two cases for BCL2L1. Most of the genes evaluated were downregulated in the cells cultured without stimulation in this group.

BCL2L1 was upregulated in four out of eight individuals without periodontitis after HmuY stimulation, while only one individual showed upregulation when the cells were cultured without stimulus. Similarly, three individuals showed upregulation of TNFSF10, FAS, FASLG, CASP9, and APAF1 under HmuY challenge. In the P group, after stimulation with HmuY protein, the individual who showed upregulation in BID, BAX, CASP3, and BCL2L1 also showed indetermination in TNF, TNFSF10, FAS, FASLG, CASP9, and APAF1, differently from the cells grown without stimulus, which showed downregulation in the case of all these genes. The other individual who showed upregulation in BCL2L1 also demonstrated upregulation in TNF. The pattern of regulation changed from downregulation to indetermination in most of the cases.

### 3.2. Apoptosis-Related Gene Expression in the Extrinsic Pathway

**Figure 2:**
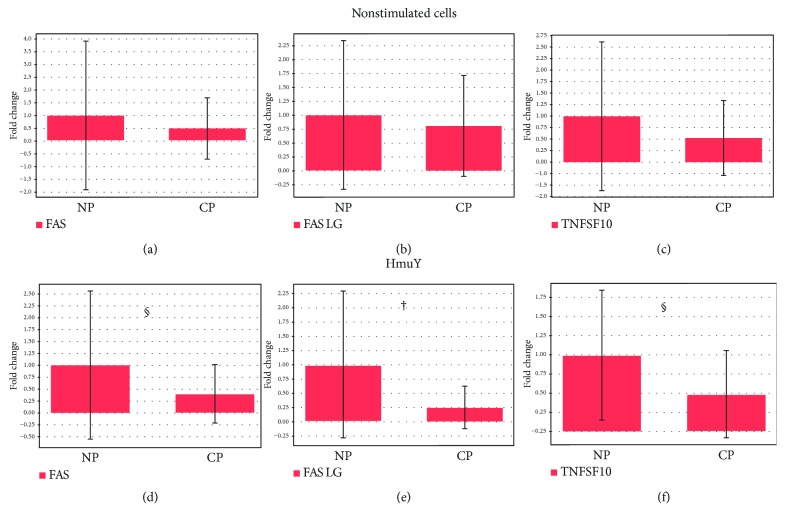
Apoptosis-related extrinsic pathway gene expression in the PBMC of the (a-c) P and NP groups without stimuli and under (d-f) *P. gingivalis* HmuY protein stimulation. (a, d) FAS (TNF receptor superfamily, member 6); (b, e) FAS LG (TNF superfamily, member 6 ligand); (c, f) TNFSF10 (tumor necrosis factor ligand superfamily-10). ^†^*p* = 0.01 and ^§^*p* < 0.1.

PBMC from the P group showed downregulation and lower levels for FAS ligand compared to those from the NP group (*p* = 0.01), as observed in Figure 2, under stimulation with *P. gingivalis* HmuY protein. Under the same conditions, the downregulation of FAS and TNFSF10 in the P group, but not statistically significant (*p* = 0.09; *p* = 0.06, respectively) (Figures 2(d) and 2(f)), was observed. When PBMCs were cultured without stimulus, no statistically significant difference was found for FAS (*p* = 0.31), FAS ligand (*p* = 0.43), or TNFSF10 (TRAIL) (*p* = 0.22) expression between the P and NP groups (Figures 2(a)–2(c)).

### 3.3. Apoptosis-Related Gene Expression in the Intrinsic Pathway

**Figure 3:**
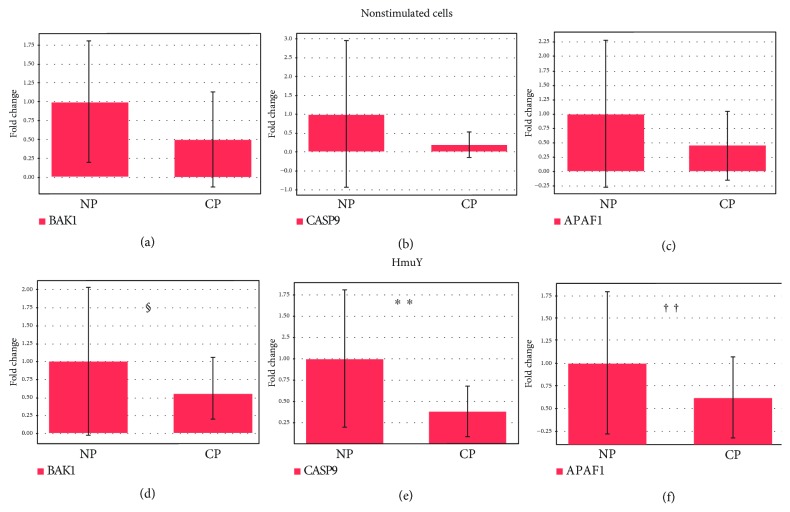
Apoptosis-related intrinsic pathway gene expression in the PBMC of the (a-c) P and NP groups without stimuli and under (d-f) *P. gingivalis* HmuY protein stimulation. (a, d) BAK1 (BCL2-antagonist/killer 1); (b, e) CASP9 (caspase 9); (c, f) APAF1 (apoptotic peptidase activating factor-1). ^∗∗^*p* = 0.01, ^††^*p* = 0.05, and ^§^*p* < 0.1.

The downregulation and lower levels of CASP9 (*p* = 0.003) and APAF1 (*p* = 0.048) in PBMC in the P group compared to PBMC from the NP group were observed, when the cells were cultured under stimulation of *P. gingivalis* HmuY protein (Figures 3(e) and 3(f)). Downregulation was found in the P group for the expression of BAK1, compared to the NP group, but this difference is not statistically significant (*p* = 0.085) (Figure 3(a)). There was no statistically significant difference between the P and NP groups in the BAK1 (*p* = 0.35), CASP9 (*p* = 0.25), and APAF1 (*p* = 0.6) expression for unstimulated PBMC (nonstimulated cells) (Figures 3(d)–3(f)).

### 3.4. Caspase 7 Gene Expression

**Figure 4:**
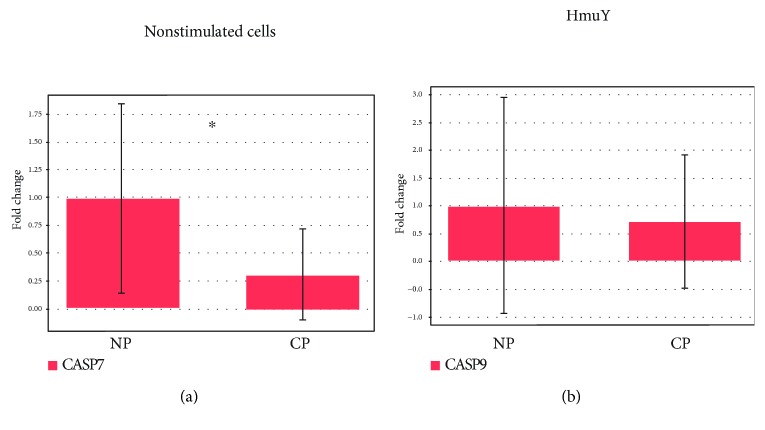
Caspase 7 expression in the PBMC of the (a) P and NP groups without stimulation (nonstimulated cells) and under (b) *P. gingivalis* HmuY protein stimulation. Caspase 7 (apoptosis-related cysteine peptidase). ^∗^*p* = 0.05.

PBMC from the P and NP groups stimulated with *P. gingivalis* HmuY protein (HmuY) did not demonstrate a statistically significant difference for caspase 7 (*p* = 0.40) (Figure 4(a)). However, there was downregulation and lower levels for caspase 7 in unstimulated cells from the P group in comparison to the NP group (*p* = 0.05) (Figure 4(b)).

## 4. Discussion

The most common outcome in inflammatory/infectious conditions is cell death by necrosis [26]. However, previous studies have demonstrated both induction and inhibition of apoptosis mechanisms in different cells types through the challenge of oral microorganisms and/or their components [21, 27–31]. It was demonstrated that the upregulation of apoptosis-related proteins occurred in gingival epithelial cells challenged with *Porphyromonas gingivalis*, *Tannerella forsythia*, and *Treponema denticola* [32]. In addition, *P. gingivalis* products seem to be able to induce the expression of genes related to apoptosis in osteogenic bone marrow stromal cells [33] and THP-1 cells [34]. The potential of *P. gingivalis* HmuY protein to interfere with cell death mechanisms in PBMC has been shown previously [16, 17].

In this study, the involvement of genes encoding apoptosis-related proteins that participate in the intrinsic and extrinsic pathways of cell death that were expressed differently in the PBMC of the P and NP groups was demonstrated. In the extrinsic pathway, HmuY was able to downregulate the expression of FASL in individuals with periodontitis. In the intrinsic pathway, *P. gingivalis* HmuY protein downregulated the caspase 9 and APAF1 expression in cells from diseased individuals. Thus, it seems that *P. gingivalis* uses this protein to suppress apoptosis of the defense host cells through both pathways, corroborating previous studies that proposed a role of delaying apoptosis [16] and increasing the production of BCL-2 [17] by HmuY.

Several studies have demonstrated many factors to suppress or inhibit apoptosis, showing the dependence of genes which encode pro- and antiapoptotic proteins in the balance of this process [35, 36]. In the presence of a bacterial challenge, it is possible to observe DNA damage associated with apoptosis and expression of p53, BCL-2 [37], FAS, FASL, and active caspase 3 in gingival tissues [38]. *P. gingivalis* can induce apoptosis in human gingival epithelial cells through the increase of FASL expression and the increase of the gene transcription mediated by NF*κ*B [39]. The oligomerization of APAF1, induced by its binding to cytochrome c, forms an apoptosome, a known structure that recruits and activates a caspase initiator, caspase 9, which, in turn, cleaves and activates caspase effector caspase 3 and caspase 7, leading to apoptosis [40].

Regarding FAS, TNFSF10 (extrinsic pathway), and BAK1 (intrinsic pathway), consistently with the results shown above, the downregulation of mRNA expression was observed in cells from individuals with periodontitis under HmuY stimulus. However, the differences found between this group and individuals without the disease showed borderline *p* values. Therefore, these results must be confirmed in studies using a larger sample size to improve the statistical power. These findings showed that *P. gingivalis* HmuY protein may impair or retard the process of apoptosis by downregulation of genes that initiate the extrinsic and intrinsic signaling pathways in periodontitis. It is known that in the extrinsic pathway, apoptosis can be induced by cell surface receptors, such as FAS, TNFR1, and TNFSF10 [41–43], while intrinsic apoptotic stimuli can activate BAX and BAK and, consequently, cause mitochondrial outer membrane permeabilization [40].

In a previous study, using *P. gingivalis* lipopolysaccharide, the upregulation of mRNA for IL-1*β* and FAS ligand was identified in a mouse periodontitis model [44]. Furthermore, the expression of mRNA for FAS and FAS-L in human gingival epithelial cells (CEGH) was upregulated by heat-killed *P. gingivalis*, and programmed cell death was induced [39].

In the assessment of caspase 7, downregulation in the mRNA expression in cells from periodontitis individuals cultured without stimulus in comparison to cells from control individuals has been demonstrated. This difference was not observed when the cells were cultured in the presence of *P. gingivalis* HmuY protein. Caspase 7 has been found in inflammatory conditions induced by bacterial infections to be involved as an effector molecule in apoptosis responsible for inducing cell detachment and ROS production in a redundant way with caspase 3 [45, 46].

Thereby, the negative modulation through the inhibition of the apoptotic process can enable *P. gingivalis* to infect host cells and provide an escape mechanism from the host immune system. These findings suggest that this keystone pathogen can reduce or inhibit PBMC apoptosis through downregulation of mRNA genes which are involved in the intrinsic and extrinsic pathways of early proapoptotic signaling in programmed cell death under *P. gingivalis* HmuY protein stimulation in individuals with periodontitis. This process could lead to continuous production of proinflammatory cytokines and chemokines in the microenvironment of the inflamed periodontal tissue, improving survival and chemotaxis of cells involved in immune defense mechanisms. It can result in a chronic inflammatory state of destruction of periodontal tissues and possibly in the establishment of a dysbiotic process in affected individuals.

We are aware that a limitation of the present study is the relatively small sample size, which suggests the need for further studies with larger numbers of participants to establish the role of the *P. gingivalis* HmuY protein in inflammatory and molecular mechanisms of cell death, providing new insights into the immunopathogenesis of periodontitis. In addition, the experiments were carried out using PBMCs and cannot be extrapolated to the microenvironment of the periodontal lesion, in which resident cells play a major role in the pathogenesis of the disease *in vivo*.

In conclusion, *P. gingivalis* HmuY protein might contribute to the survival of PBMC in periodontitis by regulating the transcriptional profile of genes involved in apoptosis, such as FASL, caspase 9, APAF1, FAS, TNFSF10 (TRAIL), and BAK1. It can lead to the perpetuation and aggravation of the inflammatory condition of the periodontal structures through the release of more inflammatory mediators which contribute to the breakdown of tissues.

## Figures and Tables

**Table 1:** Clinical findings of patients with periodontitis (P) and nonperiodontitis (NP) subjects.

	NP	P	*p* value
Number of men/women	9/17	7/13	0.958
Age (years) (mean ± SD)	37.92 ± 10.8	41.95 ± 9.7	0.565
% Bleeding on probing (mean ± SD)	8.45 ± 10.7	37.97 ± 16.9	0.028
% Probing depth ≥ 4 (mean ± SD)	1.23 ± 1.84	14.93 ± 8.91	0.001
% Clinical attachment loss ≥ 3 (mean ± SD)	19.60 ± 15.53	57.04 ± 17.25	0.001

SD = standard deviation, *p* = significance level (*p* ≤ 0.05).

## Data Availability

The data underlying the findings of this study are available in the online repository: https://repositorio.ufba.br/ri/bitstream/ri/21723/1/TESE%20PAULO%20CIRINO.pdf [47].
